# Exhaustive Exercise and Post-exercise Protein Plus Carbohydrate Supplementation Affect Plasma and Urine Concentrations of Sulfur Amino Acids, the Ratio of Methionine to Homocysteine and Glutathione in Elite Male Cyclists

**DOI:** 10.3389/fphys.2020.609335

**Published:** 2020-12-15

**Authors:** Thomas Olsen, Ove Sollie, Eha Nurk, Cheryl Turner, Fredrik Jernerén, John L. Ivy, Kathrine J. Vinknes, Matthieu Clauss, Helga Refsum, Jørgen Jensen

**Affiliations:** ^1^Department of Nutrition, Institute of Basic Medical Sciences, University of Oslo, Oslo, Norway; ^2^Department of Physical Performance, Norwegian School of Sport Sciences, Oslo, Norway; ^3^National Institute of Health Development, Tallinn, Estonia; ^4^Department of Pharmacology, University of Oxford, Oxford, United Kingdom; ^5^Department of Pharmaceutical Biosciences, Uppsala University, Uppsala, Sweden; ^6^Department of Kinesiology and Health Education, The University of Texas at Austin, Austin, TX, United States

**Keywords:** protein supplementation, carbohydrate supplementation, exercise, performance, methylation, oxidative stress

## Abstract

Plasma and tissue sulfur amino acid (SAA) availability are crucial for intracellular methylation reactions and cellular antioxidant defense, which are important processes during exercise and in recovery. In this randomized, controlled crossover trial among eight elite male cyclists, we explored the effect of exhaustive exercise and post-exercise supplementation with carbohydrates and protein (CHO+PROT) vs. carbohydrates (CHO) on plasma and urine SAAs, a potential new marker of methylation capacity (methionine/total homocysteine ratio [Met/tHcy]) and related metabolites. The purpose of the study was to further explore the role of SAAs in exercise and recovery. Athletes cycled to exhaustion and consumed supplements immediately after and in 30 min intervals for 120 min post-exercise. After ~18 h recovery, performance was tested in a time trial in which the CHO+PROT group cycled 8.5% faster compared to the CHO group (41:53 ± 1:51 vs. 45:26 ± 1:32 min, *p* < 0.05). Plasma methionine decreased by ~23% during exhaustive exercise. Two h post-exercise, further decline in methionine had occured by ~55% in the CHO group vs. ~33% in the CHO+PROT group (p_group_ × _time_ < 0.001). The Met/tHcy ratio decreased by ~33% during exhaustive exercise, and by ~54% in the CHO group vs. ~27% in the CHO+PROT group (p_group_ × _time_ < 0.001) post-exercise. Plasma cystathionine increased by ~72% in the CHO group and ~282% in the CHO+PROT group post-exercise (p_group_ × _time_ < 0.001). Plasma total cysteine, taurine and total glutathione increased by 12% (*p* = 0.03), 85% (*p* < 0.001) and 17% (*p* = 0.02), respectively during exhaustive exercise. Using publicly available transcriptomic data, we report upregulated transcript levels of skeletal muscle *SLC7A5* (log_2_ fold-change: 0.45, FDR:1.8^e−07^) and *MAT2A (*log_2_ fold-change: 0.38, FDR: 3.4^e−0.7^) after acute exercise. Our results show that exercise acutely lowers plasma methionine and the Met/tHcy ratio. This response was attenuated in the CHO+PROT compared to the CHO group in the early recovery phase potentially affecting methylation capacity and contributing to improved recovery.

## Introduction

**Figure 1:**
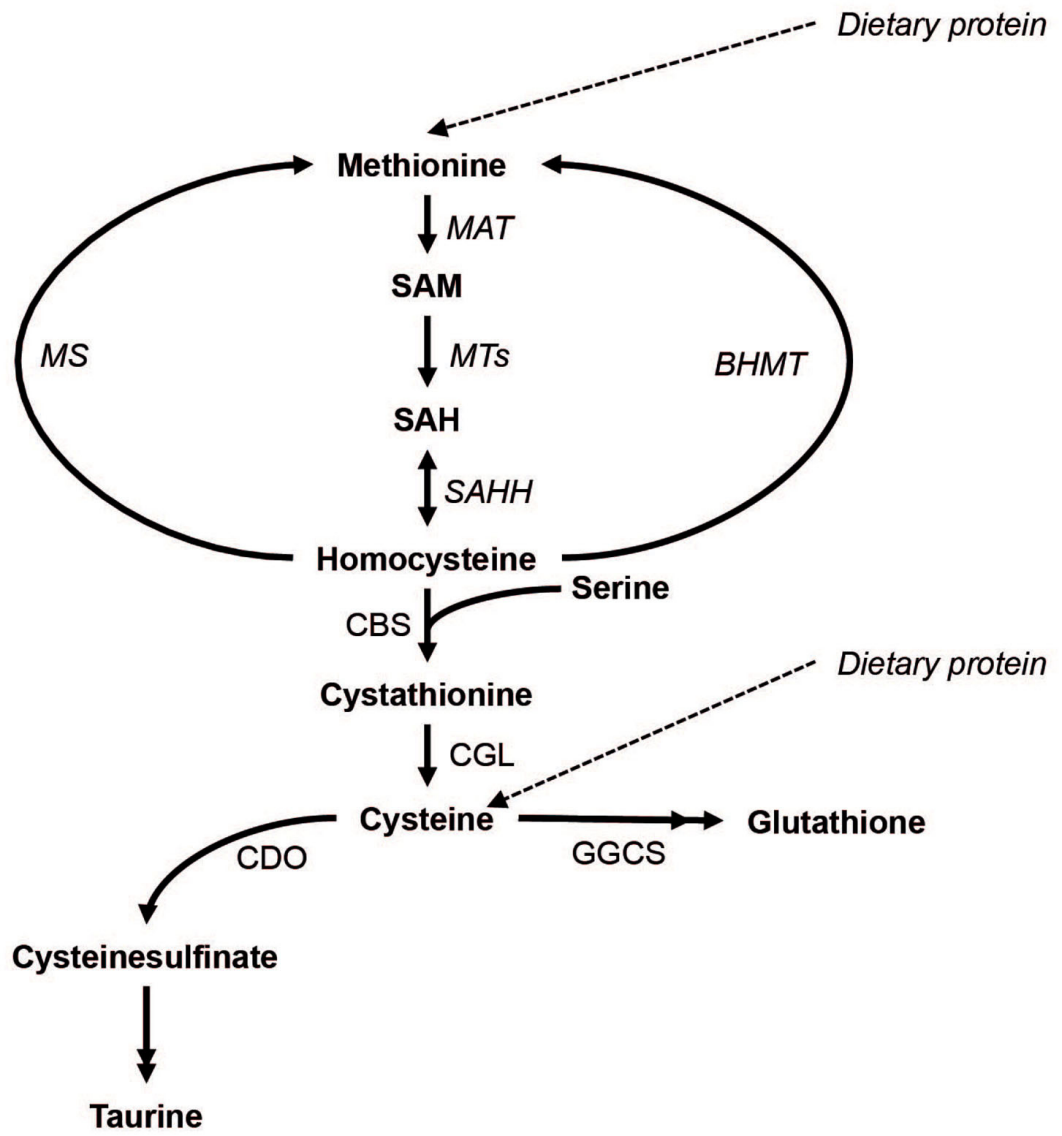
The metabolism of methionine and its downstream metabolites. Double arrow heads indicate omitted steps for simplicity. MAT, Methionine adenosyltransferase; MTs, methyltransferases; MS, methionine synthase; BHMT, betaine-homocysteine methyltransferase; SAHH, S-adenosylhomocysteine hydrolase; CBS, cystathionine beta-synthase; CGL, cystathionine gamma-lyase; CDO, cysteine dioxygenase; GGCS, gamma-glutamylcysteine synthase.

Sulfur amino acids (SAAs) include proteinogenic methionine and cysteine as well as non-proteinogenic sulfur compounds such as homocysteine, cystathionine, taurine, and the tripeptide glutathione (Brosnan and Brosnan, 2006). The metabolism of SAAs involves the conversion of methionine to S-adenosylmethionine and subsequently to homocysteine (transmethylation), which in turn can undergo re-methylation to methionine or irreversible transsulfuration (see Figure 1 for details). In transmethylation, methyl groups are transferred from S-adenosylmethionine to methyl acceptors including DNA and histones, with implications for epigenetic regulation of gene expression. In transsulfuration, homocysteine is used to irreversibly form cystathionine and subsequently cysteine, which in turn can be converted to taurine or glutathione.

Studies using exhaustive exercise and short recovery periods ranging from ~4 to 18 h have implied a role of amino acid repletion in the recovery phase for subsequent performance (Williams et al., 2003; Rustad et al., 2016; Sollie et al., 2018; Dahl et al., 2020), although there is still some debate on the effectiveness of protein supplementation for recovery and performance in endurance exercise (Kloby Nielsen et al., 2020). SAA metabolism and availability may be implicated in these processes through several important mechanisms. Hypo- and hypertmethylation of DNA and histones are thought to be implicated in tissue response to acute and long-term exercise (Barres et al., 2012; Voisin et al., 2015; Seaborne et al., 2018; Hunter et al., 2019; Landen et al., 2019; Turner et al., 2019), and are dependent on availability of plasma and cellular methionine concentrations (Mentch et al., 2015). Tissue consumption of methionine for methylation reactions have been demonstrated in exercising mice (Riberio et al., 2018) and has been linked to observed post-exercise increases in plasma total homocysteine (tHcy) in humans (Deminice et al., 2016). In addition to being a potential marker of methylation reactions, elevated tHcy may impair endothelial nitric oxide synthesis (Stühlinger et al., 2001) with potential adverse effects on recovery and performance (Kingwell, 2000). Finally, SAA availability is crucial for synthesis of the major systemic antioxidant glutathione, a process that is activated by reactive oxidative species (ROS) (Banerjee et al., 2003; Stipanuk, 2004) formed during exercise (Radak et al., 2008, 2013).

To further elucidate the role of SAAs in exercise and recovery, we analyzed plasma and urine SAAs and related metabolites from a previously published double-blind, randomized cross-over trial in 8 elite cyclists, where performance was demonstrated to dramatically improve after co-ingestion of carbohydrates and protein (CHO+PROT) compared to carbohydrates (CHO) alone (Sollie et al., 2018). The present study was exploratory and made use of biological material and data that has been analyzed *post-hoc*. The specific aims were to evaluate exercise effects on (1) plasma concentrations of all SAAs (Figure 1) and serine, and (2) the ratio of methionine to tHcy (Met/tHcy), a potential marker of methylation capacity (Hooshmand et al., 2019; Calderon-Larranaga et al., 2020), and (3) total glutathione concentrations (tGSH). Therefore, we additionally evaluated whether co-ingestion of CHO+PROT vs. CHO during the early recovery phase influenced plasma and urine concentrations of SAA, Met/tHcy, tGSH and serine concentrations post-exercise.

## Methods

### Participants

Detailed methods for pre-screening of the participants, power calculations, and protocols have been published previously (Sollie et al., 2018). Eight male elite cyclists were enrolled in the study. All athletes had participated in national and/or international competitions. They were informed of any potential risk involved in the study before providing their written informed consent. The Regional Ethics Committee of Norway reviewed the study but concluded that the project was outside their mandate (health sciences) and provided a letter of exemption (Available at the Regional Ethic Committee Public Records, ref: 2011/1298), judging that the project did not require ethical approval by the committee according to Norwegian law and the Health Research Act. The study was conducted according to the Declaration of Helsinki and Norwegian law. Written informed consent was obtained from all participants. Characteristics of the study participants including age, height, weight, watts and maximal oxygen consumption (VO_2_max) determined at pre-screening are given in Table 1.

**Table 1:** Baseline characteristics of the participants at first visit (*n* = 8)^a^.

	**Mean**	**SD**
Age, *y*	22.7	3.50
Height, *cm*	182	4.04
Weight, *kg*	79.4	7.93
VO_2_max, ml*kg^−1^*min^−1^	74.7	4.01
VO_2_max, l*min^−1^	6.03	0.84
W_max_, *watt*	464	34.0

a *Baseline characteristics of the participants. SD, standard deviation*.

### Study Design

**Figure 2:**
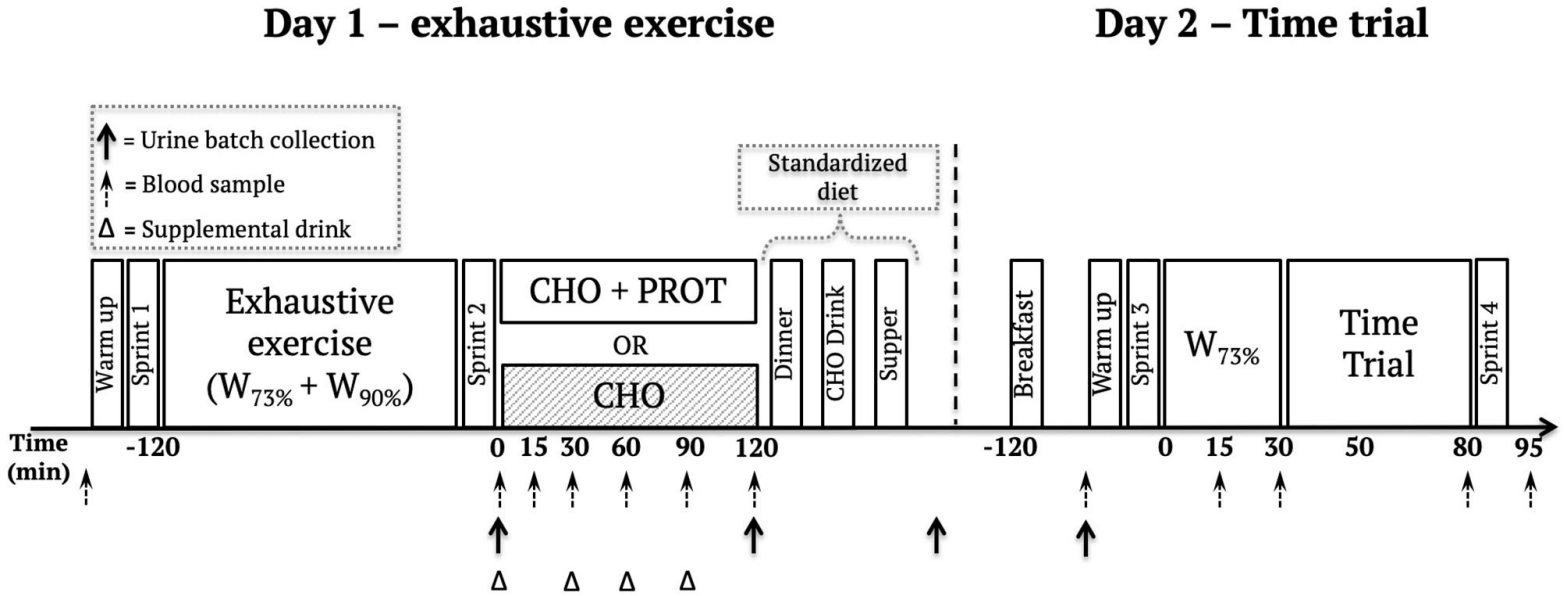
Overview of the study design.

The study was a double-blind, randomized, crossover trial, in which participants underwent two experimental interventions, both spanning two testing days. Crossover was separated by at least six days. Athletes recorded their dietary intake and exercise for 24 h prior to the start of the first experimental intervention and were instructed to follow the same diet and exercise prior to crossover. They were further instructed to do very light or no exercise 24 h prior to the intervention and to repeat this strategy before the second intervention. A simplified overview of the study can be found in Figure 2.

### Exhaustive Exercise

The first testing day consisted of an exhaustive cycling exercise bout followed by 120 min recovery intervention where the participants were provided CHO+PROT or CHO recovery drinks. The exercise session was initiated with a warm-up and the exhaustive exercise bout started with 30 min cycling at 73% of VO_2_ max (W_73%_), followed by 5 min recovery and a 10 s sprint. After 5 min recovery, intervals of 20 min at 73% of VO_2_ max were performed with 5 min recovery between each interval until athletes could not maintain their pre-determined cadence. After exhaustion, participants recovered for 5 min and completed repeated 1 min intervals at a power output corresponding to 90% of VO_2_ max (W_90%_) followed by 1 min recovery, until voluntary exhaustion. After the 1 min intervals, athletes recovered for 5 min before completing a second 10 s sprint.

### Blood Sampling

For serial blood sampling 0, 15, 30, 60, 90, and 120 min after exhaustion, a catheter (18GA, BD Venflon Pro, New Jersey, USA) was inserted into the antecubital vein. The venous blood samples were taken into 6.5 mL BD Vacutainer tubes (New Jersey, USA) containing K_2_EDTA, placed on ice and centrifuged for 10 min at 4°C (2500 g). Plasma was stored at−80°C until analysis. Urine was collected in plastic containers in consecutive 4 batches: (1) From baseline and until the end of exhaustive exercise, (2) 0-120 min after exhaustive exercise, (3) from 120 min after exhaustive exercise until midnight, (4) from midnight until arrival the following day. Volume for each period was measured and a 12 mL sample frozen at−20°C for analysis.

### Supplement Drinks and Standardized Diet

Supplement drinks were consumed after the first blood sample, and every 30 min for the first 120 min. The isovolumetric flavor-matched supplements (7.06 mL · kg^−1^ · h^−1^) contained 1.2 g carbohydrate · kg^−1^ · h^−1^ (CHO) or 0.8 carbohydrate · kg^−1^ · h^−1^ + 0.4 g whey protein · kg^−1^ · h^−1^ (CHO+PROT). The carbohydrate portion of the drinks consisted of equal amounts of maltodextrin and glucose. The supplement was provided at fixed intervals because one drink providing the total protein intake would likely result in excessive amino acid oxidation and urea excretion in line with previous reports on optimal doses to maximize post-exercise muscle protein synthesis (Moore et al., 2009; Churchward-Venne et al., 2020). Detailed composition of the diets is presented in Supplementary Table 1. The athletes followed a standardized diet in the period between the exhaustive exercise at day 1 and until the time trial (TT) ~18 h later. The diet was custom made and pre-packaged consisting of dinner (2 h post-exhaustive exercise) an evening carbohydrate drink (~4 h post-exhaustive exercise), supper (~6 h post-exhaustive exercise) and breakfast (~16 h post-exhaustive exercise). Total nutrient intake for the full 18 h recovery period was 7.29 g carbohydrate/kg, 1.08 g protein/kg and 0.77 g fat/kg in the CHO group, and 6.49 g carbohydrate/kg, 1.88 g protein/kg, and 0.77 g fat/kg. The diets were isoenergetic at 169 kJ/kg and differed only in carbohydrate and protein content due to the nutrient composition of the supplements for the first two hours post-exercise.

### Time Trial

~18 h after exhaustion, subjects underwent warm-up and a pre-loaded TT consisting of 30 min cycling at a fixed intensity (73% of VO_2_ max) before a 5 min recovery. Then, subjects underwent a TT to complete a specific amount of mechanical work, corresponding to 30 min at a workload corresponding to 100% of VO_2max_ (Work output (kJ) = Power at VO_2max_ (W) ^*^ 1800 s). Blood samples were obtained at the start of the TT and then at 15, 30 and 70 min after start of TT as well as 15 min post-TT.

### Biochemical Analyses

Blood samples were stored at −80°C until analysis. Storage time for the blood samples until analyses were ~3 y. All analytes were measured by liquid chromatography-tandem mass spectrometry (LC-MS/MS). Plasma methionine, total tHcy, total cysteine (tCys), cystathionine, total glutathione (tGSH) and serine were analyzed using a modified version of a previously described method (Antoniades et al., 2009). Deuterium-labeled isotopes were added to plasma as internal standards, followed by reduction of disulphides using dithioerythritol and then protein precipitation using perchloric acid. The acidic extracts were diluted with an aqueous solution of heptanesulfonic acid prior to LC-MS/MS. Chromatographic separation was achieved with an aqueous solution of formic acid [0.05%] and methanol gradient mobile phase. Positive mode multiple reaction monitoring was used for detection. Linear calibration curves of the peak area ratios of analyte and internal standard were used for quantification. Plasma taurine was extracted and analyzed separately. Deuterium-labeled taurine isotope was added to plasma as an internal standard, followed by protein precipitation using cold methanol. The extracts were diluted with an aqueous solution of formic acid [0.5%] and heptafluorobutyric acid [0.3%] prior to LC-MS/MS. Chromatographic separation was achieved with an aqueous solution of formic acid [0.5%] and heptafluorobutyric acid [0.3%] and acetonitrile gradient mobile phase. Positive mode multiple reaction monitoring was used for detection. Linear calibration curves of the peak area ratios of analyte and internal standard were used for quantification.

### External Datasets

To further explore mechanisms by which exercise can affect SAA metabolism, we used external data from public datasets to assess expression of genes involved in SAA metabolism and uptake in skeletal muscle. Data are available from the meta-analysis published by Pillon et al. (2020) and were analyzed with the companion web-based application (www.metamex.eu). To achieve comparability with the current data, we assessed the effects of exercise on expression levels in skeletal muscle of young and middle-aged, lean healthy, physically active or athletic adults (*n* = 48) who provided biopsies either immediately or 1 h after acute exercise. Accession numbers of the external datasets are GSE120862, GSE107934, GSE71972, GSE71972, GSE33603, and GSE44818.

### Statistics

Normality was assessed with visual evaluation of quantile-quantile plots. Values are expressed as mean ± standard deviation (SD) unless otherwise specified. Unadjusted effects of exhaustive exercise were assessed with paired samples t-tests (post-exercise vs. pre-exercise). We used body weight changes from before to after exercise as a proxy for changes in blood volume. To assess body weight-adjusted effects of exhaustive exercise on plasma concentrations, we constructed mixed models with the plasma amino acid as the outcome and a time variable (before and after exercise) as exposure. Body weight and a random term for subject ID accounting for within-subject correlation was also added to this model. Linear mixed model regression was also used to determine group differences in the plasma amino acid response to the supplement drinks, with group, time and group × time used as fixed effects and subject ID as random effect to account for correlated observations within subjects. Thus, the interaction effects in this model indicate group differences in response to the supplement drinks.

Tests were considered significant when *p* < 0.05. Statistical analyses were performed using the “base,” “stats,” and “lme4” packages for R versions 4.0.0-4.0.2 (the R Foundation for Statistical Computing, Vienna, Austria). Plots were made using the “ggplot2” package.

## Results

On the exhaustion test, participants cycled a mean (SD) 112 (9) min and 108 (6) min at 73% VO_2_max before the CHO and CHO+PROT interventions, respectively. At the TT the following morning the CHO+PROT group cycled 8.5% faster compared to the CHO group (41:53 ± 1:51 vs. 45:26 ± 1:32 min, *p* < 0.05). Metabolic parameters for the exhaustive exercise bout are presented in Supplementary Table 2, and a detailed description of the metabolic response during exhaustive exercise and the TT has been published elsewhere (Sollie et al., 2018).

**Figure 3:**
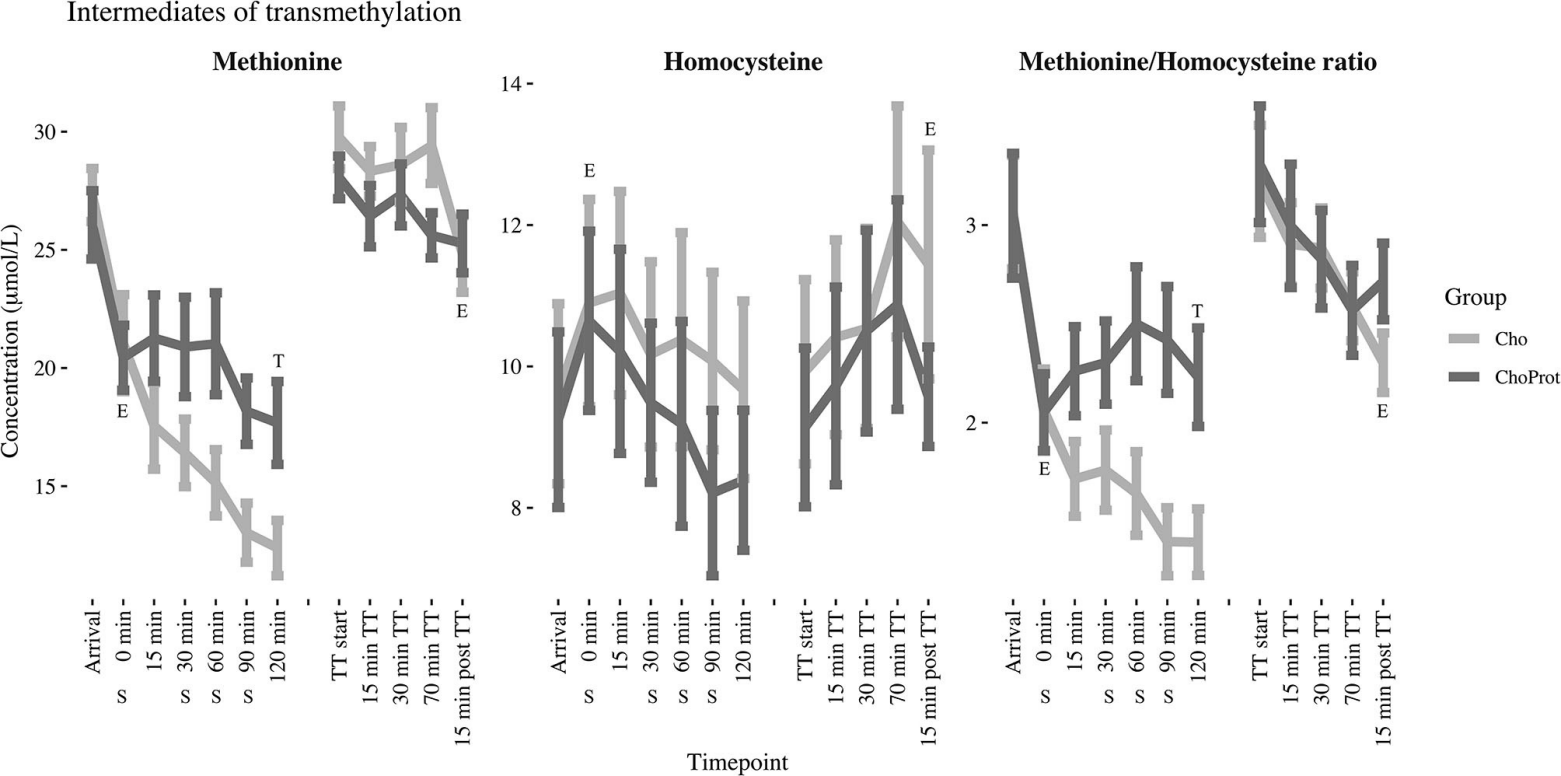
Changes in estimated marginal mean (standard error) methionine, total homocysteine and their ratio from arrival up to 120 min post exhaustive exercise. Derived from the linear mixed models. E, significant effect of exercise; T, significant group difference between groups over time; CHO, carbohydrate supplement drink; CHO+PROT, carbohydrate and protein supplement drink; TT, time trial; S, supplement drink.

**Figure 4:**
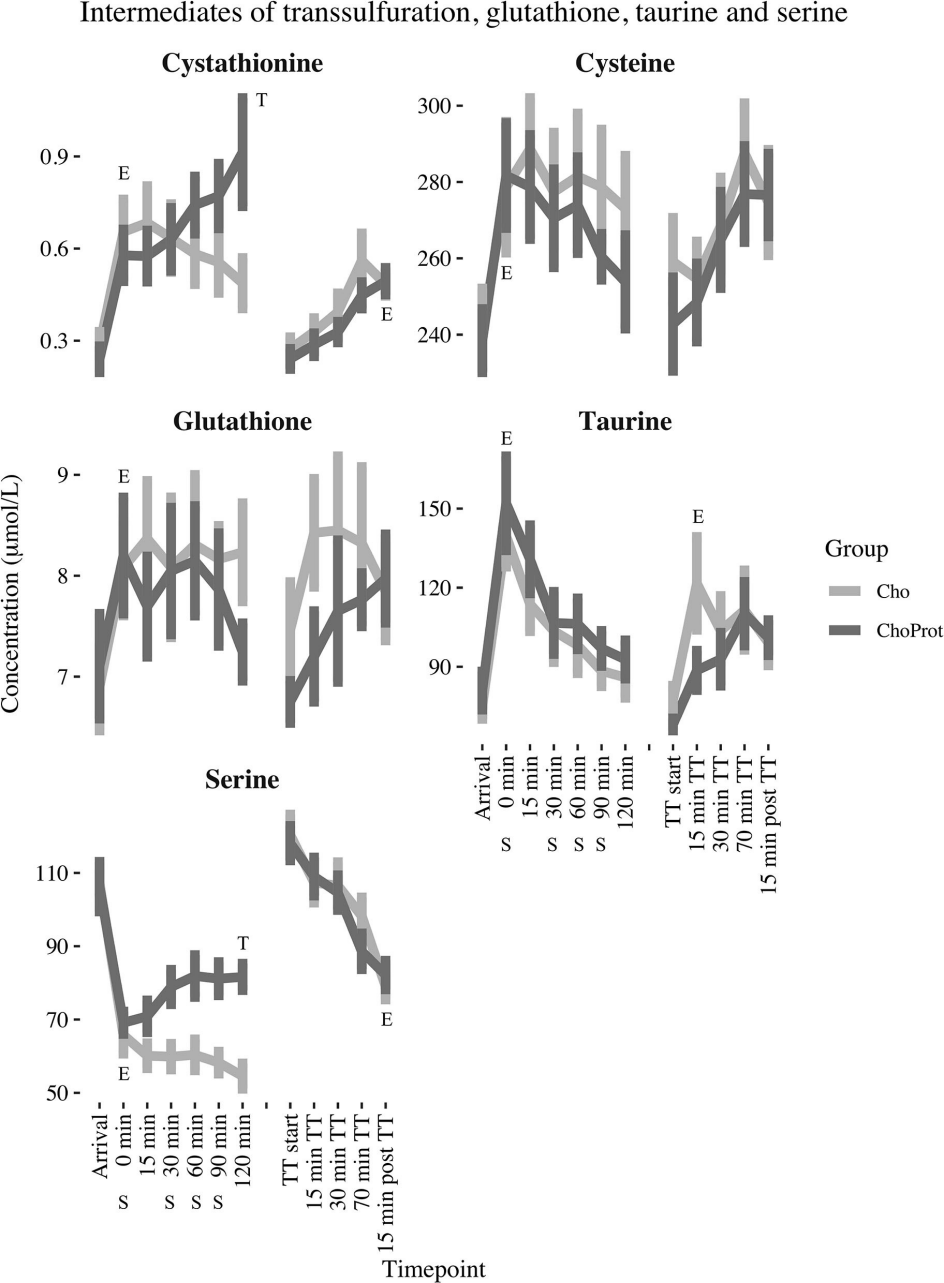
Changes in estimated marginal mean (standard error) cystathionine, cysteine, taurine, glutathione and serine from arrival up to 120 min post exhaustive exercise. E, significant effect of exercise; T, significant group difference between groups over time; CHO, carbohydrate supplement drink; CHO+PROT, carbohydrate and protein supplement drink; TT, time trial; S, supplement drink.

The acute effect of exhaustive exercise and the TT on plasma concentrations of SAAs, cystathionine, tGSH and serine are presented in Table 2 and Figures 3, 4. Linear mixed model regression estimates for the post-exhaustive exercise response and the TT are presented in Supplementary Table 3. Concentrations of the amino acids at all timepoints are presented in Supplementary Tables 4, 5. Plasma methionine decreased by ~23% during exhaustive exercise. During the first 120 min of recovery, methionine was further reduced by ~55% in the CHO group, whereas a decrease of ~33% was observed in the CHO+PROT group (*p* for group × time interaction < 0.001). The following morning, plasma methionine had returned to baseline values. During the TT, methionine decreased slightly in both groups. Plasma tHcy increased by ~15% during exhaustive exercise, with no differences between groups 120 min post-exercise. The Met/tHcy ratio, an indicator of methylation capacity, decreased by ~33% during exhaustive exercise, and had further decreased by ~54% in the CHO group 120 min post-exercise, whereas a decrease of ~27% was observed in the CHO+PROT group (p for group × time interaction < 0.001). Cystathionine increased by ~131% during exercise and subsequently decreased to ~72% relative to baseline 120 min post-exercise in the CHO group, whereas a continued increase was observed in the CHO+PROT group reaching ~282% relative to baseline concentrations 120 min post-exercise (p for group × time interaction < 0.001). Plasma tCys, taurine and tGSH increased by 12, 85, and 17%, respectively during exhaustive exercise, with no differences between groups in response to CHO or CHO+PROT ingestion 120 min post-exercise. Finally, because serine condenses with homocysteine during transsulfuration we assessed the exercise and supplement effects on plasma concentrations of serine. Serine decreased by ~39% during exhaustive exercise and had further decreased by ~49% in the CHO group 120 min after exercise whereas the decrease was attenuated in the CHO+PROT group (p for group × time interaction < 0.001). Similar exercise patterns were observed on the TT the following day.

**Table 2:** Sulfur amino acids and serine concentrations at arrival and post-exercise and time trial^a, b^.

	**Arrival**		**Post-EXH**		
**Analytes, *μmol/L***	**Mean**	**SD**	**Mean**	**SD**	***P***
Methionine	27.3	3.18	21.0	5.81	0.014
tHcy	9.25	3.51	10.65	3.59	0.012^c^
Met/tHcy	3.06	0.81	1.40	0.48	<0.001
Cystathionine, *nmol/L*	283	129	654	297	<0.001
tCys	241	30.4	278	48.5	0.030
Taurine	75.1	13.9	139	32.9	<0.001
tGSH	6.90	1.22	8.08	1.34	0.020
Serine	107	10.1	65.8	14.3	<0.001
	**Pre-TT**		**15 min post-TT**		
Methionine	28.06	2.55	25.27	3.51	0.012
tHcy	9.14	3.17	9.57	1.99	0.07
Met/tHcy	3.31	0.83	2.71	0.55	0.012
Cystathionine, *nmol/L*	240.75	92.93	493.71	122.24	<0.001
tCys	242.78	34.65	276.59	30.66	0.14
Taurine	68.13	6.51	101.00	18.76	0.024
tGSH	6.75	0.59	7.97	1.24	0.33
Serine	106	19.1	69.1	8.66	0.016

a *Plasma concentrations of amino acids upon arrival and immediately after cycling to exhaustion. Data are from the CHO visit of all participants*.

b *Post-EXH, post-exhaustive exercise; tHcy, total homocysteine; tCys, total cysteine; tGSH, total glutathione*.

c *In a model adjusting for body weight changes to exhaustive exercise, the effect on total homocysteine was attenuated and no longer significant*.

To account for changes in blood volume due to dehydration we adjusted the statistical analyses for body weight before and after exhaustive exercise. Except for a slight attenuation of the exhaustive exercise effect on plasma concentrations of tHcy (β_post−exhaustive exercise vs. before_ [standard error]: 1.24 [0.85] *p* = 0.15), all other effects of exhaustive exercise were robust to this adjustment (data not shown).

The effect of the supplements on total urinary excretion of the SAAs and tGSH are presented in Table 3. Total urinary excretion of cystathionine and tGSH was significantly higher in the CHO+PROT group compared to the CHO group.

**Table 3:** Total urinary excretion of sulfur amino acids and serine during the testing period^a, b^.

	**CHO**		**CHO+PROT**		
**Urinary amino acids, *μmol/18h***	**Mean**	**SD**	**Mean**	**SD**	***p***
Methionine	2.96	1.0	2.89	0.92	0.81
tHcy	4.73	3.66	4.71	2.77	0.97
Cystathionine	22.3	16.7	32.8	18.5	<0.01
tCys	300	78.6	300	68.1	0.97
Taurine	1443	641	2124	1324	0.11
tGSH	1.0	0.3	1.3	0.5	0.03
Serine	523	88.5	575	117	0.10

a *Total excretion of amino acids from immediately after the exhaustive exercise test and up to the performance test*.

b *CHO, carbohydrate supplement drink; CHO+PROT, carbohydrate and protein supplement drink; tHcy, total homocysteine; tCys, total cysteine; tGSH, total glutathione*.

To further explore relevant mechanisms underlying the acute exercise response, we used data from five publicly available datasets with skeletal muscle biopsies (Pillon et al., 2020). We used data from lean, healthy, physically active individuals that underwent an acute aerobic exercise test and explored effects on expression levels of genes related to methionine uptake and metabolism. mRNA transcripts of the large neutral amino acid transporter (LAT1) were significantly upregulated after an acute exercise bout (*SLC7A5*: Meta-analysis restricted maximum likelihood log_2_ fold-change: 0.45, FDR: 1.8^e−07^). In addition, transcript levels of *MAT2A* which catalyze the formation of S-adenosylmethionine were significantly upregulated (log_2_ fold-change: 0.38, FDR 3.4^e−07^). Transcript levels for all genes involved in SAA metabolism are given in Supplementary Table 6.

## Discussion

### Principal Findings

**Figure 5:**
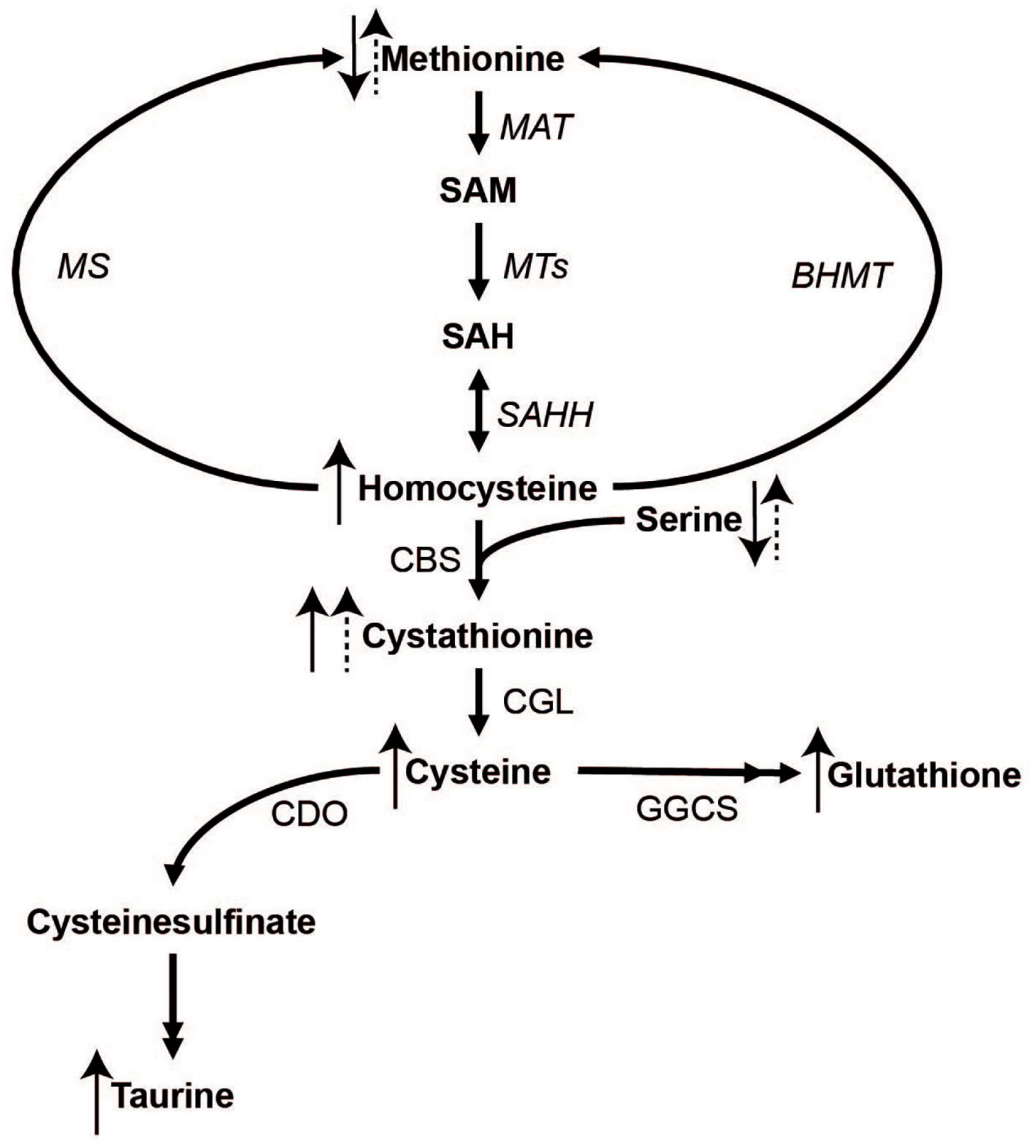
The metabolism of sulfur amino acids with arrow indicators of change in plasma concentrations during exhaustive exercise and the supplementation period. Solid arrows represent changes after exhaustive exercise, dashed arrows represent responses to carbohydrate + protein supplementation vs. carbohydrate supplementation.

In this exploratory study, we show that plasma concentrations of methionine and serine decrease after exhaustive exercise, whereas plasma concentrations of the other SAAs increased. Main findings are summarized in Figure 5. In the following paragraphs we discuss our findings in light of the wider literature and outline potential underlying mechanisms. Due to the exploratory nature of the study, we note that the potential mechanisms are meant to aid interpretation and generate hypotheses that may be pursued in future studies, and not provide proof or definite explanations.

Plasma methionine continued to decrease during the recovery period after CHO ingestion and was reduced by 55% after 120 min recovery. Importantly, the methionine decrease was attenuated in the CHO+PROT group, stabilizing at 17.7 μmol/L compared to 12.4 μmol/L in the CHO group after 120 min of recovery. A similar observation was made for the Met/tHcy ratio, a plasma indicator of methylation capacity, which was 2.23 in the CHO+PROT group vs. 1.40 in the CHO group after 120 min of recovery. These novel findings are in agreement with our previous study (Rustad et al., 2016) and provide additional data showing the changes in SAA availability and metabolism during exercise and recovery and subsequent exercise performance. The present study also indicates that CHO+PROT ingestion exerts profound effects on intracellular methylation capacity, which may in part explain the benefits of protein intake in the immediate exercise recovery phase (Williams et al., 2003; Ferguson-Stegall et al., 2011b; Rustad et al., 2016; Sollie et al., 2018; Dahl et al., 2020).

A 23% decrease in plasma methionine was observed after exhaustive exercise, whereas plasma tHcy, tCys and cystathionine increased. With regards to the exercise-induced decrease in methionine, Lee et al. reported that an acute exercise bout at 70% of VO_2_max significantly reduces plasma methionine concentrations of sedentary males (Lee et al., 2018). Plasma methionine concentrations have also been found to decline after 120 min of cycling at 60% of VO_2_max (Galloway et al., 2008), and after cycling to exhaustion at 72% VO_2_max (Rustad et al., 2016). However, limited or no decrease in plasma methionine was reported for less extensive exercise (Forslund et al., 2000; Venta et al., 2009). In contrast to plasma methionine, tHcy concentrations have consistently been reported to increase after acute exercise (Deminice et al., 2016), whereas muscle methionine levels have been found to increase in exercising humans and animals (Blomstrand and Saltin, 1999; Ishikura et al., 2013). Taken together, the decrease in plasma methionine and increase in its downstream metabolites suggest that SAA metabolism is activated by exercise above a certain intensity threshold, possibly due to increased tissue uptake and metabolism and in response to oxidative stress.

#### SAA Response to Supplement Drinks

The continued decrease in plasma methionine observed after CHO ingestion in the present study parallels the reductions in plasma BCAA reported in previous studies (Rustad et al., 2016; Sollie et al., 2018), suggesting that insulin response to the CHO supplement may have facilitated the decrease in methionine due to increased tissue uptake. Interestingly, one euglycemic hyperinsulinemic clamp study in healthy men demonstrated that plasma methionine decreased after insulin was raised (Tessari et al., 2005). Compartmental modeling based on isotope dilution techniques in the same study demonstrated a six-fold increase in intracellular transmethylation reaction kinetics when insulin was raised. This particular finding suggests that the observed decline in plasma methionine and the Met/tHcy ratio in the CHO group may in part be due to increased insulin affecting methionine uptake and intracellular transmethylation reactions. Changes in insulin in the present study was published in the original paper (Sollie et al., 2018), and showed that insulin increased similarly in both groups, if not slightly more in the CHO+PROT group. The abovementioned activation of methionine metabolism after exhaustive exercise may therefore have become reinforced by insulin in the CHO group. In the CHO+PROT group, this response may have been prolonged due to the persistent ingestion of methionine and other SAA via the protein supplement.

### Potential Mechanisms

#### Methionine, Homocysteine, Methylation, and Exercise

The Met/tHcy ratio may be used as an indicator of methylation capacity as suggested previously (Hooshmand et al., 2019; Calderon-Larranaga et al., 2020). The observed decrease in methionine and increase in tHcy post-exhaustive exercise may thus reflect increased transmethylation in which DNA, histones and other macromolecules are methylated in response to exercise. Indeed, one animal experiment showed that plasma methionine availability is an important determinant of intracellular methylation capacity (Mentch et al., 2015), and post-exercise elevations in tHcy is thought to reflect increased methyl flux in tissues (Riberio et al., 2018). It has been reported that acute exercise induced immediate hypomethylation of several genes in skeletal muscle biopsies obtained from healthy individuals (Barres et al., 2012). These findings tended to be reversed 3 h post-exhaustive exercise, implying compensatory re-methylation of promoter regions in the recovery phase. In addition, methylome studies in humans show that both hypermethylation and hypomethylation occur following exercise regimens (Turner et al., 2019), and that specific promoters of exercise-responsive genes such as *PGC1*α can be both hypomethylated (Barres et al., 2012) and hypermethylated (Lochmann et al., 2015) after exercise. Notably, plasma methionine was reduced by more than 50% 120 min post-exhaustive exercise in the CHO group of the present study. Considering the dependency of DNA and histone methyltransferases on plasma methionine availability (Petrossian and Clarke, 2011) and the positive effects of insulin on tissue methionine uptake and transmethylation reactions (Tessari et al., 2005), increased tissue uptake and flux through transmethylation reactions in response to exercise and CHO ingestion may be potential mechanisms underlying our findings with respect to the Met/tHcy ratio.

It is currently not known whether Met/tHcy reflects cellular methylation capacity similar to the ratio of S-adenosylmethionine to S-adenosylhomocysteine, although our observations seem to reflect effects of exercise on S-adenosylmethionine, S-adenosylhomocysteine and homocysteine in animals leading to increase flux through the transmethylation pathway (Riberio et al., 2018). Unfortunately, determination of S-adenosylmethionine and S-adenosylhomocysteine in plasma require specialized methods for sample collection (Olsen et al., 2018), which was not performed in the present study. Measurement of these analytes in addition to tissue biopsies for methylome screening should be considered in future studies in order to further unravel the role of methionine and SAA metabolism in exercise, recovery and performance.

#### Transsulfuration, Glutathione, and Exercise

The transsulfuration pathway, which catalyzes the conversion of homocysteine to cystathionine and cysteine, is activated by oxidative stress and cellular S-adenosylmethionine (Brosnan and Brosnan, 2006). Notably, methionine and S-adenosylmethionine increase in plasma shortly after intake of a methionine-rich meal (Olsen et al., 2020), and considering the importance of their plasma availability for intracellular processes (Mentch et al., 2015), intake of methionine-rich protein sources such as whey, may induce intracellular transsulfuration. Supporting this notion, cystathionine, the product of the rate-limiting step of transsulfuration, increased dramatically in plasma after exhaustive exercise in the present study and a continued increase was observed throughout the 120 min recovery period in the CHO+PROT group. In addition, serine, which condenses with homocysteine to produce cystathionine, decreased sharply after exhaustive exercise. One explanation for this hypothesized increase in transsulfuration is that this pathway provides substrates for synthesis of the major antioxidant glutathione, which may be in demand during and after exhaustive exercise (Radak et al., 2008, 2013). In the present study, plasma tGSH increased after exhaustive exercise, which is partly in line with previous studies. One study showed that oxidized glutathione increased in the plasma of cyclists following a mountain stage (Aguilo et al., 2005), whereas it decreased in plasma of cyclists after a flat race stage (Cordova et al., 2015). Although we did not observe increased plasma concentrations of tGSH during either intervention, urinary excretion of both cystathionine and tGSH was elevated in the CHO+PROT group the following morning. Overall, these results indicate altered SAA metabolism in favor of transsulfuration, possibly to provide sufficient glutathione in response to exercise. Although we have insufficient data on the redox systems, we note that homocysteine re-methylation is inhibited by oxidative stress whereas both reactions of transsulfuration is activated by it (Joseph and Loscalzo, 2013; Sbodio et al., 2019). Future studies assessing the role of exercise-induced oxidative stress on SAA and the relationship with redox systems should aim to measure reduced and oxidized fractions of glutathione in blood, hippuric acid in urine and components of the thioredoxin and nicotinamide dinucleotide (NAD) systems. Importantly, methionine metabolism can provide substrates for oxidative phosphorylation and NAD metabolism has been suggested to link methionine metabolism with the tricarboxylic acid cycle in a recent study (Lozoya et al., 2018).

#### Taurine and Exercise

There was an ~85% increase in plasma concentrations of taurine after exhaustive exercise whereas no effects were observed between the supplement groups. Taurine production is activated when cysteine increases and is one of the main pathways for cysteine degradation catalyzed by cysteine dioxygenase in the liver (Stipanuk, 2004; Brosnan and Brosnan, 2006). The hypothesized increase in transsulfuration and subsequent cysteine production may thus have contributed to increased plasma concentrations of taurine. In addition, an antioxidant role for taurine has been suggested in exercise (Spriet and Whitfield, 2015) and hepatic taurine production and release may be increased in response to exercise-induced oxidative stress. However, experimental studies have shown that overexpression of cysteine dioxygenase depletes glutathione pools (John et al., 2007) rendering this mechanism implausible in the context of exercise-induced oxidative stress where glutathione may be in demand. Considering that skeletal muscle contains large amounts of taurine, another potential explanation could be that some taurine leaks from muscle during exercise (Spriet and Whitfield, 2015). However, human skeletal muscle taurine contents remain stable during 120 min of exercise at 60% VO_2_max (Galloway et al., 2008), which further complicates interpretation. It should be noted that the taurine response may depend on intensity and duration of exercise, and that the exhaustive exercise bout in our study may have been more physically demanding compared to earlier trials (Galloway et al., 2008). There were no effects of the supplements on taurine concentrations in the recovery phase. Without further data, it is difficult to assess the importance of changes in taurine concentrations on performance the following day.

#### Expression of Genes in SAA Metabolism in Exercise

Co-ingestion of CHO+PROT may improve consecutive-day performance (Rustad et al., 2016; Sollie et al., 2018) and VO_2_max over time (Ferguson-Stegall et al., 2011a) by influencing the transcription of several genes in skeletal muscle (Rowlands et al., 2011). Indeed, data included in this article from external sources (Pillon et al., 2020) showed that genes related to amino acid uptake (*SLC7A5*/*SLC3A2*) and formation of S-adenosylmethionine (*MAT2A*), the primary methyl donor, were upregulated after acute exercise. These data support our hypothesis that methionine and SAA metabolism including methylation reactions may be involved in post-exercise recovery. Genes in transsulfuration were not increased in skeletal muscle after exercise. This finding is not surprising since transsulfuration as well as glutathione and taurine synthesis mainly takes place in the liver and because regulation of several enzymes occur on the protein level (Stipanuk, 2004; Brosnan and Brosnan, 2006). These data were included with the purpose of supporting our findings with more information beyond plasma and urine, as we do not have tissue available for analysis. We note that these data are not intended to be explanatory for our findings as they were generated from different studies with differing design.

### Strengths and Limitations

The main strength of this study is its randomized crossover design, which is essential for minimizing bias and the exercise studies were conducted in a rigorous manner. The CHO group in this study is believed to serve as an adequate control group. However, the lack of a pure placebo condition may be considered a limitation e.g., considering the potential mediating effects of the insulin response which would not be expected in a placebo arm. The small sample size and lack of female participants limits statistical power and generalizability, respectively, and in line with this we emphasize that the aim of the study was strictly exploratory and not inferential, highlighting the need for future studies specifically designed to assess SAA metabolism in exercise. For example, plasma concentrations can reflect several processes including externalization, internalization, flux and dietary intake, and thus there is a need for studies that are specifically designed to investigate SAA exchange between compartments and organs during exercise. In addition, it should be mentioned that the concentrations of SAA also depend on other unmeasured factors such as choline, betaine and vitamins B6, B9, and B12, which may have affected the reported results. The SAAs were measured in stored samples. However, even at −25°C, the stability of most of the measured analytes are generally acceptable (Hustad et al., 2012). Another consideration is that the plasma concentrations can be affected by dehydration, as genes in methionine metabolism are affected by changes in blood volume (Hoffmann et al., 2013). However, adjusting analyses for body weight loss during exhaustive exercise only minimally affected the results. Finally, it would have been useful to measure total excretion of the amino acids before the testing period to assess the effects of exercise on total excretion vs. resting conditions.

## Conclusions

In this exploratory study, we show that plasma concentrations of methionine and an indicator of methylation capacity, Met/tHcy, decrease after exhaustive exercise, and continue to decrease if protein is not provided in the immediate recovery phase. Together with the observed increase in plasma concentrations of other SAAs, this suggests that methionine metabolism may be activated by exercise with potential ramifications for recovery and subsequent performance if post-exercise protein is not provided. Our findings underline that ingestion of whole-protein sources after exercise may be preferable to supplements with individual or a few amino acids, consistent with other results. Given the observed changes in SAA concentrations with the potential to influence SAA metabolism, our results suggest that the beneficial effect of whole protein could be related to its content of methionine. Future studies should be carried out in a larger sample and aim to characterize mechanisms involved in methylation processes and redox status that might impact exercise recovery, and how post-exercise protein nutrition modifies the effects of exercise on cellular methylation capacity. Preferably, tissue data should be collected in order to further address the relevance of the proposed mechanisms.

## Data Availability Statement

The raw data supporting the conclusions of this article will be made available by the authors, without undue reservation.

## Ethics Statement

Ethical review and approval was not required for the study on human participants in accordance with the local legislation and institutional requirements. The patients/participants provided their written informed consent to participate in this study.

## Author Contributions

OS, JI, and JJ designed the study and collected the data. TO and EN performed statistical analyses. TO, JI, and JJ drafted the manuscript. CT, FJ, and HR were responsible for biochemical analyses. KV and MC made intellectual contributions. All authors read, revised and approved the final version of the manuscript.

## Conflict of Interest

The authors declare that the research was conducted in the absence of any commercial or financial relationships that could be construed as a potential conflict of interest.
