# Supplementary material for: Exhaustive Exercise and Post-exercise Protein Plus Carbohydrate Supplementation Affect Plasma and Urine Concentrations of Sulfur Amino Acids, the Ratio of Methionine to Homocysteine and Glutathione in Elite Male Cyclists
Source: Front Physiol. 2020 Dec 15;11:609335. doi: 10.3389/fphys.2020.609335 (PMC7769812; doi:10.3389/fphys.2020.609335)
Supplement: Supplementary file 1 [file Data_Sheet_1.docx]

| Supplemental Table 1: Composition of the standardized meals after the intervention | | | | | | |
| --- | --- | --- | --- | --- | --- | --- |
|  | **Time** | **Time after EXH** | **Carbohydrate**  **(g·kg^-1^)** | **Protein**  **(g·kg^-1^)** | **Fat**  **(g·kg^-1^)** | **Energy content**  **(kJ·kg^-1^)** |
| Dinner | ~ 17.00 | ~ 2 h | 1.4 | 0.47 | 0.29 | 39.01 |
| Evening carbohydrate drink | ~ 19.00 | ~ 4 h | 1.2 | 0 | 0 | 20.40 |
| Supper | ~ 21.00 | ~ 6 h | 0.97 | 0.29 | 0.24 | 30.54 |
| Breakfast | ~ 07.00 | ~ 16 h | 1.32 | 0.32 | 0.24 | 37.00 |

| **Supplemental Table 2: Metabolic parameters during exhaustive exercise^1^** | | |
| --- | --- | --- |
|  | Mean | SD |
| Average watt, *watt* | 298 | 34.0 |
| Time to exhaustion, *min* | 111 | 26.8 |
| VO_2max_, *l/min^-1^* | 4.51 | 0.65 |
| Sprints, *n* | 13 | 7 |
| Total carbohydrate oxidation, *grams* | 371 | 101 |
| Glucose start, mmol/L | 4.4 | 0.78 |
| Glucose end, mmol/L | 3.5 | 1.11 |
| ΔGlucose (end-start), mmol/L | -1.0 | 1.61 |
| Lactate start, mmol/L | 1.2 | 0.30 |
| Lactate end, mmol/L | 2.9 | 1.01 |
| ΔLactate (end-start), mmol/L | 1.7 | 1.10 |

**^1^**Metabolic parameters during exhaustive exercise. Data are from the 1^st^ visit of all participants. Abbreviations; SD, standard deviation

| **Supplemental Table 3: Linear mixed model estimates for changes in amino acids between groups 0-120 min after the intervention and after the time trial^1, 2^** | | | | |
| --- | --- | --- | --- | --- |
| **Exhaustive exercise and 120 recovery** |  |  |  |  |
|  | Time | Group | Time^*^Group | p*_interaction_* |
| Methionine | -2.22 ± 0.21 | -1.54 ± 1.33 | 1.21 ± 0.30 | < 0.001 |
| tHcy | -0.07 ± 0.08 | -0.05 ± 0.47 | -0.21 ± 0.11 | 0.053 |
| Met/tHcy | -0.23 ± 0.03 | -0.16 ± 0.18 | 0.17 ± 0.04 | < 0.001 |
| Cystathionine | 8.43 ± 12.7 | -253 ± 78.6 | 85.5 ± 18.2 | < 0.001 |
| tCys | 3.40 ± 1.60 | 3.46 ± 9.98 | -2.86 ± 2.25 | 0.210 |
| Taurine | -2.99 ± 2.06 | 10.9 ± 12.8 | -0.53 ± 2.89 | 0.856 |
| tGSH | 0.16 ± 0.11 | 0.35 ± 0.55 | -0.16 ± 0.13 | 0.202 |
| Serine | -1.50 ± 0.58 | -2.30 ± 3.89 | 4.37 ± 0.82 | < 0.001 |
| **Time trial** |  |  |  |  |
| Methionine | -0.81 ± 0.30 | -3.76 ± 3.44 | 0.19 ± 0.43 | 0.66 |
| tHcy | 0.43 ± 0.11 | -1.06 ± 1.67 | 0.04 ± 0.15 | 0.79 |
| Met/tHcy | -0.20 ± 0.04 | 0.07 ± 0.56 | 0 ± 0.05 | 0.96 |
| Cystathionine | 71.0 ± 10.5 | 20.1 ± 163.5 | -6.99 ± 14.8 | 0.64 |
| tCys | 6.27 ± 2.00 | -43.6 ± 31.2 | 3.34 ± 2.83 | 0.24 |
| Taurine | 4.16 ± 3.31 | -60.0 ± 51.6 | 4.42 ± 0.94 | 0.35 |
| tGSH | 0.10 ± 0.11 | -2.59 ± 1.74 | 0.17 ± 0.16 | 0.28 |
| Serine | -9.27 ± 11.1 | 2.66 ± 14.6 | -0.44 ± 1.32 | 0.74 |
| ^1^Model estimates for change in plasma amino acids between groups at 0-120 minutes after the intervention. The interaction term indicates the time-dependent change in each group (protein + carbohydrate group vs carbohydrate group)  ^2^Abbreviations: Met/tHcy, ratio of methionine to total homocysteine; tHcy, total homocysteine; tCys, total cysteine; tGSH, total glutathione | | | | |

| **Supplemental Table 4: Amino acid concentrations before, and after exhaustive exercise and recovery after exercise^1^** | | | | | | | | | | | | | | |
| --- | --- | --- | --- | --- | --- | --- | --- | --- | --- | --- | --- | --- | --- | --- |
| Carbohydrate group | | | | | | | | | | | | | | |
|  | Arrival | | 0 min | | 15 min | | 30 min | | 60 min | | 90 min | | 120 min | |
| Amino acids, *μmol/L* | Mean | SD | Mean | SD | Mean | SD | Mean | SD | Mean | SD | Mean | SD | Mean | SD |
| Methionine | 27.32 | 3.18 | 21.05 | 5.81 | 17.55 | 5.18 | 16.41 | 4.04 | 15.14 | 3.97 | 13.03 | 3.53 | 12.38 | 3.32 |
| tHcy | 9.61 | 3.60 | 10.90 | 4.15 | 11.04 | 4.07 | 10.17 | 3.71 | 10.38 | 4.29 | 10.08 | 3.55 | 9.67 | 3.55 |
| Met/tHcy | 3.07 | 0.81 | 2.06 | 0.60 | 1.72 | 0.54 | 1.76 | 0.57 | 1.64 | 0.60 | 1.40 | 0.49 | 1.40 | 0.47 |
| Cystathionine, *nmol/L* | 283.09 | 129.54 | 654.49 | 297.87 | 683.66 | 338.35 | 634.28 | 312.51 | 582.56 | 277.94 | 555.22 | 281.22 | 487.23 | 232.03 |
| tCys | 241.32 | 30.49 | 278.64 | 48.51 | 288.66 | 37.75 | 277.38 | 44.00 | 281.46 | 46.61 | 278.64 | 42.75 | 272.98 | 39.33 |
| Taurine | 75.17 | 13.97 | 139.62 | 32.99 | 113.78 | 29.05 | 103.55 | 33.34 | 98.11 | 29.78 | 88.42 | 16.41 | 85.96 | 21.92 |
| tGSH | 6.90 | 1.22 | 8.08 | 1.34 | 8.37 | 1.62 | 8.08 | 1.96 | 8.31 | 1.96 | 8.17 | 0.93 | 8.23 | 1.38 |
| Serine | 107 | 10.1 | 65.8 | 14.3 | 60.1 | 9.64 | 59.9 | 9.91 | 60.4 | 11.9 | 58.3 | 8.45 | 54.6 | 9.78 |
| Carbohydrate + protein group | | | | | | | | | | | | | | |
| Methionine | 26.06 | 4.09 | 20.44 | 3.88 | 21.27 | 5.16 | 20.89 | 5.95 | 21.03 | 6.10 | 18.18 | 3.98 | 17.68 | 4.96 |
| tHcy | 9.25 | 3.51 | 10.65 | 3.59 | 10.21 | 4.08 | 9.49 | 3.18 | 9.19 | 4.10 | 8.21 | 3.31 | 8.39 | 2.80 |
| Met/tHcy | 3.05 | 0.89 | 2.05 | 0.55 | 2.26 | 0.64 | 2.30 | 0.60 | 2.50 | 0.82 | 2.42 | 0.76 | 2.23 | 0.70 |
| Cystathionine, *nmol/L* | 239.58 | 119.80 | 577.86 | 238.21 | 575.46 | 236.04 | 630.14 | 289.29 | 741.46 | 263.69 | 771.16 | 297.79 | 913.95 | 498.44 |
| tCys | 238.44 | 23.44 | 281.69 | 38.89 | 278.70 | 38.61 | 270.50 | 36.34 | 273.94 | 35.54 | 260.40 | 17.05 | 253.83 | 34.66 |
| Taurine | 80.96 | 20.42 | 152.01 | 50.42 | 130.77 | 36.62 | 106.64 | 33.37 | 106.28 | 27.32 | 96.96 | 18.80 | 92.78 | 20.51 |
| tGSH | 7.10 | 1.47 | 8.20 | 1.63 | 7.69 | 1.41 | 8.05 | 1.78 | 8.15 | 1.54 | 7.86 | 1.58 | 7.24 | 0.80 |
| Serine | 106 | 19.1 | 69.1 | 8.66 | 70.1 | 12.3 | 78.9 | 13.3 | 81.9 | 16.2 | 81.1 | 12.8 | 81.6 | 10.2 |

^1^Abbreviations: Met/tHcy, ratio of methionine to total homocysteine; tHcy, total homocysteine; tCys, total cysteine; tGSH, total glutathione, SD; standard deviation.

| **Supplemental Table 5: Amino acid concentrations before, during and after the time trial^1^** | | | | | | | | | | |
| --- | --- | --- | --- | --- | --- | --- | --- | --- | --- | --- |
| Carbohydrate group | | | | | | | | | | |
|  | TT Start | | 15 min TT | | 30 min TT | | 70 min TT | | 15 min post TT | |
| Amino acids, *μmol/L* | Mean | SD | Mean | SD | Mean | SD | Mean | SD | Mean | SD |
| Methionine | 29.76 | 3.75 | 28.33 | 2.96 | 28.60 | 4.48 | 29.41 | 4.53 | 24.83 | 4.59 |
| tHcy | 9.93 | 3.68 | 10.41 | 3.89 | 10.54 | 4.00 | 12.05 | 4.62 | 11.44 | 4.58 |
| Met/tHcy | 3.22 | 0.80 | 2.90 | 0.60 | 2.88 | 0.57 | 2.59 | 0.49 | 2.30 | 0.43 |
| Cystathionine, *nmol/L* | 272.75 | 108.42 | 328.75 | 126.11 | 393.13 | 172.84 | 561.09 | 249.71 | 483.43 | 104.84 |
| tCys | 259.14 | 32.44 | 254.59 | 27.78 | 268.48 | 35.95 | 287.56 | 37.03 | 274.63 | 39.08 |
| Taurine | 77.25 | 15.67 | 121.63 | 49.77 | 103.63 | 37.36 | 111.50 | 42.61 | 99.14 | 24.33 |
| tGSH | 7.46 | 1.34 | 8.43 | 1.51 | 8.45 | 2.08 | 8.33 | 2.13 | 7.84 | 1.37 |
| Serine | 120 | 15.7 | 107 | 15.5 | 107 | 18.2 | 98.3 | 14.3 | 79.0 | 9.97 |
| Carbohydrate + protein group | | | | | | | | | | |
| Methionine | 28.06 | 2.55 | 26.43 | 3.67 | 27.33 | 3.71 | 25.61 | 2.70 | 25.27 | 3.51 |
| tHcy | 9.14 | 3.17 | 9.73 | 3.95 | 10.50 | 4.04 | 10.88 | 4.19 | 9.57 | 1.99 |
| Met/tHcy | 3.31 | 0.83 | 3.00 | 0.88 | 2.83 | 0.70 | 2.57 | 0.64 | 2.71 | 0.55 |
| Cystathionine, *nmol/L* | 240.75 | 92.93 | 286.75 | 106.18 | 328.13 | 95.34 | 447.65 | 122.03 | 493.71 | 122.24 |
| tCys | 242.78 | 34.65 | 248.43 | 28.96 | 264.83 | 35.73 | 276.85 | 35.61 | 276.59 | 30.66 |
| Taurine | 68.13 | 6.51 | 88.63 | 21.08 | 92.88 | 28.44 | 110.13 | 34.16 | 101.00 | 18.76 |
| tGSH | 6.75 | 0.59 | 7.20 | 1.27 | 7.65 | 1.98 | 7.76 | 0.75 | 7.97 | 1.24 |
| Serine | 118 | 13.3 | 109 | 14.7 | 105 | 13.4 | 88.6 | 13.8 | 82.1 | 11.1 |

^1^Abbreviations: Met/tHcy, ratio of methionine to total homocysteine; tHcy, total homocysteine; tCys, total cysteine; tGSH, total glutathione, SD; standard deviation.

**Supplemental Table 6: Expression of genes involved in sulfur amino acid metabolism after exercise. Data are based on external datasets (GSE120862, GSE107934, GSE71972, GSE71972, GSE33603 and GSE44818).**

| Gene | Function | Log_2_ fold change after acute exercise | Restricted maximum likelihood FDR |
| --- | --- | --- | --- |
| *SLC7A5* | Cellular amino acid uptake. Component of the large amino acid transporter 1. | 0.45 | 1.8^e-07,*^ |
| *SLC3A2* | Cellular amino acid uptake. Component of the large amino acid transporter 1. | 0.06 | 0.23 |
| *MAT2A* | Adenosylation of methionine to form S-adenosylmethionine | 0.38 | 3.4^e-07,*^ |
| *MAT2B* | Regulatory subunit of MAT2A | -0.07 | 0.15 |
| *MTR* | B12 and folate-dependent re-methylation of methionine to form homocysteine | -0.03 | 0.57 |
| *CBS* | First step of transsulfuration | -0.13 | 0.25 |
| *CTH* | Second step of transsulfuration | -0.03 | 0.71 |
| *GCLC* | First step of glutathione synthesis, catalytic subunit, | -0.03 | 0.58 |
| *GCLM* | First step of glutathione synthesis, regulatory subunit | 0.00 | 0.98 |
| *GSS* | Second step of glutathione synthesis | 0.05 | 0.42 |
| *CDO1* | First step of taurine synthesis from cysteine | -0.04 | 0.71 |
| *CSAD* | Second step of taurine synthesis from cysteine | 0.05 | 0.57 |

^*^ Indicates significance
